# Biological Properties of *Aloysia gratissima* (Gillies & Hook.) Tronc. (Verbenaceae)

**DOI:** 10.1155/2022/1119435

**Published:** 2022-01-19

**Authors:** Maryelen Alijar Souza, Fernanda Petry, Letícia Vidor Morgan, Jacir Dal Magro, Liz G. Müller

**Affiliations:** ^1^Graduate Program in Environmental Sciences, Community University of Chapecó Region (Unochapecó), Chapecó 89809-900, Brazil; ^2^Area of Health Sciences, Community University of Chapecó Region (Unochapecó), Chapecó 89809-900, Brazil

## Abstract

*Aloysia gratissima* is a plant native to America, with applications in folk medicine for a wide range of diseases, such as bronchial infections, lung disorders, nervous system disorders (depression, anxiety), and inflammatory processes, among others. However, investigations about this species and its biological actions are still scarce. This literature review was carried out using articles published in the past 30 years on the PubMed, SciELO, and Web of Science platforms, with the focus on the method of extraction, chemical composition, and clinical and preclinical studies on the pharmacological properties of *A. gratissima*. We noticed that the main constituents of *A. gratissima* are guaiol, pinocamphone, *ß*-pinene, and 1,8-cineole. Additionally, preclinical studies reveal that *A. gratissima* extracts present antidepressant, anti-inflammatory, antinociceptive, antibacterial, antifungal, and virucidal effects. The results also demonstrate that there is a greater interest on the part of researchers from 2012 onwards in studying *A. gratissima* extracts with potential for possible new drugs.

## 1. Introduction

The scientific interest in compounds derived from plant extracts is associated with the knowledge of popular medicine [1]. Oils and/or extracts derived from plants can be obtained from different parts of vegetal species, including leaves, stems, roots, seeds, flowers, and fruits. [2]. It is estimated that approximately 25% of all drugs currently prescribed are derived from plants [3]. Thus, there is a notable interest from the academic community and the pharmaceutical industry in developing safer and more effective drugs to treat health disorders [4]. Additionally, natural products are active against microorganisms, such as fungi, viruses, and bacteria, responsible for several infectious diseases. These pathologies are becoming more difficult to treat due to the increasing resistance of microorganisms to antibiotics, and in some cases, there is no treatment option [5].

Among the plants found in Brazil, we highlight the genus *Aloysia* (Verbenaceae), originally from South America, containing 34 cataloged species, 12 of which can be found in Brazil [6, 7]. *Aloysia gratissima* (Gillies & Hook.) Tronc. is a shrub that can reach a maximum height of 3 m, has an irregular growth pattern, and may present thorns on the branches. Its leaves are simple and small (1–2 × 0.5–0.8 cm) [8]. The buds are strong and herbaceous, and the flowers are white, fragrant, in bunches, with intense flowering. The foliage is persistent and blooms between spring and summer. This plant is ornamental, due to the intensity of flowering and the pleasant aroma of the flowers [9, 10].

*A. gratissima*, popularly known in Brazil as “lavanda-do-brasil,” “erva-de-nossa-senhora,” “erva sagrada,” and “garupá” is widely distributed in America, occurring from the United States to Argentina. It was recently found in the central areas of the continent, northern Argentina, Brazil, Paraguay, Uruguay, and Mexico [9, 11].

In Argentina, according to Ricciardi et al. [9], *A. gratissima* is known as “Angel,” “oreganillo,” “azahar del campo,” and “niño rupá” (name recognized by the Guarani Indians). In Uruguay, it is called “cedron del monte” and in the United States, “whitebrush.” *A. gratissima* was previously classified by other botanical names, such as *Verbena gratissima* Gill. et Hook, *Aloysia lycioides* Cham., and *Lippia lycioides* (Cham.) Steudel. [7].

In Rio Grande do Sul, Brazil, *A. gratissima* is popularly used mainly for headaches, “nerve problems, disorders of the digestive and respiratory systems such as colds and bronchitis,” and inflammatory pain [11–13]. In Santa Catarina, Brazil, its use was observed in two locations—Blumenau and Guabiruba—as sedative and antidepressant [14].

The infusion of the aerial parts of this plant is widely used in Argentina as a tonic, for digestive disorders, as diaphoretic and aphrodisiac. In addition, the infusion prepared with flowers and leaves is used in Bolivia as carminative and sedative, and as antispasmodic in Paraguay and Uruguay [15].

Considering the importance of the popular use of native plants in southern Brazil and their different biological activities, this study aims to systematically examine scientific studies on the biological effects and chemical composition of *A. gratissima*.

## 2. Materials and Methods

This study used a qualitative method (literature review) and included articles published in the past 30 years. The articles were obtained using the keyword *A. gratissima* in the PubMed, SciELO, and Web of Science databases. Afterwards, the articles were selected according to the following inclusion criteria: articles with keywords in the title and/or abstract, articles about extracts of plant products or isolated compounds, and clinical or preclinical studies on pharmacological properties. In this sense, we excluded articles related to environmental toxicology and agroecology in this study.

## 3. Results and Discussion

### 3.1. Selection of Articles

**Figure 1:**
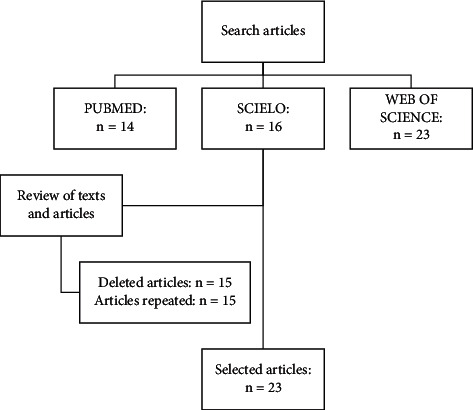
Flowchart of articles selection for the review.

In the initial search, 50 articles were identified: 14 from PubMed, 16 from SciELO, and 23 from Web of Science. In total, 15 articles were indexed in two or more databases and considered only once. After a detailed analysis regarding the titles, abstracts, and full texts, 15 articles that did not meet the search criteria were excluded. Thus, 23 articles were finally selected. The flowchart presented in Figure 1 details the progressive selection of the study.

The findings of the literature review carried out in the present study are compiled in Table 1. It was noted that there was an increase in the number of articles about *A. gratissima* after the year 2012. In some cases, there is more than one publication by the same author. This fact may be correlated to the occurrence of the plant in South America [11] (Table 1).

The methodologies adopted for extraction and the parts of the plant that were used (leaves, flowers, branches, and stems) are different between the studies. The identification of the compounds present in the oils and/or extracts was similar in all articles, with gas chromatography (GC) being the method employed by the majority of studies. It was noticed that articles about *A. gratissima* are associated with *in vitro* and *in vivo* assays. Biological properties of this species, such as virucidal [22], nematicide [33], antioxidant [29], sedative/anesthetic [6], antidepressant-like neuroprotective [14], antibacterial [28], antinociceptive, anti-inflammatory [13, 26], and antispasmodic [18], have been reported. There are no clinical studies on *A. gratissima* in the literature.

### 3.2. Chemical Composition of *Aloysia gratissima*

The chemical composition of the plant may vary according to the method of extracts and oils obtention, as well as the period of the harvest. Ricciardi et al. [9] performed the steam distillation of the aerial parts of the plant in three different harvest periods: spring (reproductive), autumn (vegetative), and summer (postreproductive) and in three different regions, such as São Lorenzo, Rio Empedrado, and Perichón. The authors identified elemene, viridiflorol, *ß*-caryophyllene, and *α*-thujone as the main constituents found in the extracts, with high levels of sesquiterpenes (34.3–51.1%). During spring, the monoterpenes content was 7.6%, while sesquiterpenes had higher results in autumn (47.2%) and summer (51.1%). The thujone isomers appeared exclusively in samples from the São Lorenzo region, and the contents varied according to the considered ontogenic stage (6.8–17.5%). The authors confirmed the presence of these compounds through IR spectral performances, with a sample/oil fraction of 1/1, showing the deformation of the carbonyl group (5.72 *μ*m). This variation of carbonyl groups was also evaluated by gas chromatography according to the time of year, resulting in higher values in spring (19.3%), autumn (13.7%), and summer (8.2%) [9].

Benovit et al. [6] used the Clevenger extraction and identified the presence of 1,8-cineole, sabinene, guaiol, and bicyclogermacrene in the pure oil extraction of *A. gratissima*. However, after the oil fractionation, the compounds E-(-)-pinocamphone, (-)-caryophyllene oxide, (-)-guaiol, and (+)-spathulenol were identified. The two above-cited studies were carried out in the Santa Maria city, Rio Grande do Sul (Brazil). Arze et al. [16] identified the constituents sabinene, *ß*-pinene, and *ß*-caryophyllene in the essential oil of *A. gratissima* collected in the Cochabamba Province of Mizque (Bolivia). The essential oil of *A. gratissima* collected in the São Carlos city, São Paulo (Brazil), studied by Trovati et al. [27], presents isopinocamphone (25.4%), limonene (15.1%), and guaiol (12.7%) as its major constituents. In this study, oxygenated monoterpenes were the main groups, representing 32.6% of the total oil; oxygenated sesquiterpenes were 24.6% of the oil, followed by monoterpene hydrocarbons (18.3%) and hydrocarbon sesquiterpenes (10.9%), among others (8.3%).

In Uruguay, Soler et al. [25, 34] report the chemical compounds present in the oil obtained from the leaves of *A. gratissima* as sabinene (30–35%), *ß*-pinene (8–10%), and *ß*-caryophyllene (∼8%), and in the flowers of the species, the main components are sabinene (19–10%) and *ß*-pinene (11–5%).

**Figure 2:**
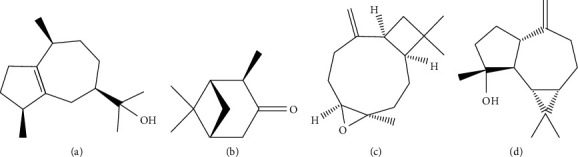
Chemical structure of the main compounds found in *Aloysia gratissima*: (a) guaiol, (b) pinocamphone, (c) caryophyllene oxide, and (d) spathulenol.

The majority of studies about the species *A. gratissima* are based on extractions by the hydrodistillation method (Clevenger). However, there are a few articles that carried out the supercritical fluid extraction of the plant. Souza et al. [13, 26] found volatile terpenic compounds in the extract of *A. gratissima* leaves obtained by supercritical fluid with carbon dioxide (SCCO_2_). The main compounds found in the supercritical extract were guaiol (18.50%) and pinocamphone (11.40%), in addition to the presence of other compounds such as (-)trans-pinocarvyl acetate (10.50%), b-cubeben (8.21%), caryophyllene (7.63%), *γ*-elemene (7.28%), caryophyllene oxide (6.76%), (-)-spatulenol (6.17%), pinocarvil acetate (5.31%), bunesol (4.67%), isopinocamphone (3.52%), myrtenol (3.24%), and humelene (2.97%) in smaller amounts. These findings agree with the chemical composition of the plant reported by other studies that used different methods of extraction. There were no significant variations in the composition of the extracts according to the pressures and temperatures operated during the extraction process. The chemical structures of the main compounds found in *A. gratissima* can be seen in Figure 2.

Santos et al. [32] evaluated the leaves and parts of the inflorescence of *A. gratissima* through hydrodistillation by Clevenger. According to the authors, the inflorescence of *A. gratissima* presented a higher content of oil (0.56%) than its leaves (0.35%). The gas chromatography-mass spectrometry (GC/MS) analysis revealed the presence of monoterpenes in the oil derived from the leaves (18.7% nonoxygenated and 22.6% oxygenated), while the essential oil of the inflorescence presented sesquiterpenes (30.4% nonoxygenated and 44.5% oxygenated), which corresponds to 74.9% of the total sesquiterpenes. Additionally, the authors [32] describe that the main compounds found in the essential oil of the leaves of *A. gratissima* were transpinocarveyl acetate (17.6%), trans-pinocamphone (16.3%), and guaiol (11.5%). Nevertheless, there is a higher content of guaiol (19.5%), germacrene B (10.5%), bulnesol (10.0%), e-caryophyllene (8.9%), and caryophyllene oxide (8.24%) in the inflorescences.

Furthermore, guaiol was identified in a hexane extract from another sample of *A. gratissima* species in Brazil, together with spathulenol [31]. Other authors have identified different proportions of guaiol in *A. gratissima* oils, such as 29.63% [19], 22.04% [13], 12.7% [27], 18.5% [13], 11.5% [24], 10.33% [13], 8.53% [35], 6.7% [6], and 2.6% [36]. These differences can be attributed to the environmental conditions (e.g., period of harvest) and the extraction method [13].

Zeni et al. [29] evaluated *A. gratissima* oil obtained by hydrodistillation, through HPLC and identified ferulic acid (11.57–86.40 mg/100 g), trans-cinnamic acid (7.97–56.92 mg/100 g), and p-coumaric acid (0.00–16.06 mg/100 g) as the most abundant phenolic acids in the studied samples. The authors also analyzed the seasonality of the oil's compounds and found that it presented higher contents of caffeic, ferulic, and t-cinnamic acids in winter when compared to the summer. However, chlorogenic acid contents were more abundant in summer.

Da Silva et al. [31] evaluated the leaves of *A. gratissima* obtained from the extraction with ethanol at room temperature, in south-central Brazil. The extract was fractionated with hexane, dichloromethane, ethyl acetate, and methanol. The chromatographic analysis was carried out on silica gel eluted with hexane solvent and the main compounds found in the fractions were guaiol, bisabolol, and spathulenes. The dichloromethane fraction was chromatographed with chloroform/methanol, demonstrating the presence of triterpenoids. The ethyl acetate extract was chromatographed with hexane/ethyl acetate and resulted in the compounds betulinic acid, oleanolic acid, and ursolic acid. Finally, the methanol fraction was chromatographed on Sephadex LH20, using water/methanol, and the compounds found in the fraction were the phenylethanoid verbascoside and arenarioside.

The essential oil of *A. gratissima* contains more than 70% hydrocarbons, of which more than half are sesquiterpenes [36]. Freires et al. [19] indicated the presence of volatile compounds, mainly oxygenated mono- and sesquiterpenes, in addition to sesquiterpenic hydrocarbons. This is particularly interesting since the pharmacological effects of plants are related to the presence of metabolites such as alkaloids, terpenes, flavonoids, and phytosterols [26].

### 3.3. Anti-Inflammatory and Antinociceptive Activities

Terpenic compounds are highly known for their pharmaceutical properties, such as antimicrobial, anti-inflammatory, and antitumor [37]. It is possible to assess the anti-inflammatory activity through the carrageenan-induced ear or paw edema assays. The assay is divided into three phases: the first phase (the first 90 minutes of the test) is related to the release of histamine and serotonin, the second phase (90–150 minutes) involves the activation of kinins, and the third phase (after 150 minutes) is related to the increased synthesis of prostaglandins in the inflamed tissue. In the last stage, there is also polyinfiltration of morphonuclear leukocytes.

Regarding the anti-inflammatory activity of *A. gratissima*, Vandresen et al. [28] demonstrated that the aqueous extract of its leaves caused a reduction of 23.6% in ear edema induced by carrageenan in mice when compared to animals treated with vehicle. This study corroborates the results found by Souza et al. [13], who demonstrated that an extract of *A. gratissima* leaves obtained by SCCO_2_ is enriched in guaiol and elicited a reduction in paw edema during the first 3 hours after the carrageenan intraplantar administration in mice. The authors suggest that the anti-inflammatory activity of the *A. gratissima* supercritical extract is not related to a decrease in the synthesis of prostaglandins but to an inhibition of the release of histamine, serotonin, and kinins. Nevertheless, further studies on the anti-inflammatory activity of this vegetal species are still necessary.

Souza et al. [26] obtained promising results with the extract of *A. gratissima* obtained by supercritical extraction with carbon dioxide. Their research demonstrated that in the acetic acid-induced abdominal writhing test in mice, the extract at 30 mg/kg (p.o.) caused a reduction of 41% in the abdominal writhes, while the dose of 10 mg/kg caused a reduction of 56%, with the number of writhes from the groups treated with both doses being statistically similar to the positive control group (indomethacin). In the formalin assay, *A. gratissima* induced antinociceptive activity on both phases of the test; hence, it is effective on both neurogenic and inflammatory pain [26]. According to Apel et al. [38], these results are probably related to its main constituents, guaiol, and spathulenol.

Souza et al. [13] also investigated the involvement of opioid receptors in the mechanism of antinociceptive action of the *A. gratissima* extract obtained by SCCO_2_ through pharmacological antagonism with naloxone in the formalin test in mice. It was observed that naloxone did not prevent the antinociceptive activity of *A. gratissima* in both phases of the formalin test, suggesting that the opioid system is unlikely to be involved in the mechanism of antinociceptive action. This result shows promising pharmacological potential for the species, since the use of opioid agents is related to the development of dependence and tolerance, in addition to side effects including sedation, constipation, and urinary retention, among others [39]. Aiming to elucidate the mechanism of action of *A. gratissima* extract, the authors demonstrated the involvement of ATP-sensitive *K* + channels by pretreating mice with glibenclamide (K(+)-channel blocker). This mechanism of antinociceptive action may be related to the terpenes present in this species [25].

### 3.4. Antimicrobial Effects

The study performed by Freires et al. [19] analyzed the bioactive fractions (BF) of *A. gratissima* against *Streptococcus mutans* in order to assess the thickness, biovolume, and morphology of biofilms treated with the BF. Briefly, the author points out that BF from *A. gratissima* had a significant effect on bacterial viability—acting as bactericide [24]—and also affected a key characteristic of the pathogenicity of *S. mutans*—the production of extracellular polysaccharides (EPS) [24]. The authors report that there was a disruption of the integrity of the biofilm; therefore, the BF may have created porosity. There was no change in the thickness, but the biomass of EPS was significantly reduced (15.63 ± 2.56 *μ*m^3^/*μ*m^2^; *p* < 0.05). One of the putative mechanisms of *A. gratissima* BF action may be related to inhibition of glycosyltransferase activity.

Santos et al. [24] evaluated the antimicrobial and antifungal activities of *A. gratissima* oil. The antimicrobial activity was evaluated against the microorganisms *Staphylococcus aureus* (ATCC 25923), *Bacillus cereus* (ATCC 11778), *Acinetobacter baumanii* (ATCC 17978), *Escherichia coli* (ATCC25922), and *Pseudomonas aeruginosa* (ATCC 27853). The antibacterial effect was evaluated by the microdilution method to determine the minimal inhibitory concentration (MIC) and minimal bactericidal concentration (MBC). The antifungal activity was evaluated using the yeasts *Cryptococcus neoformans* (ATCC 32264) and *Candida albicans* (ATCC 10231) and the filamentous fungi *Aspergillus flavus* (ATCC 9170), *Aspergillus fumigatus* (ATCC 26934), *Rhizopus* sp. (CL 35), *Microsporum canis* (C112), *Microsporum gypseum* (C115), *Trichophyton mentagrophytes* (ATCC 9972), *Trichophyton rubrum* (C137), and *Epidermophyton floccosum* (C114).

Regarding antibacterial activity, the essential oil of *A. gratissima* was active against all tested microorganisms, and *B. cereus* was the most sensitive species (MIC = 1,000 *μ*g/mL; MBC = 2,000 *μ*g/mL). Regarding the antifungal activity, the essential oil showed moderate activity against *M. gypseum* (MIC = 1,000 *μ*g/mL), *Epidermophyton flakesum* (MIC = 1,000 *μ*g/mL), *T. rubrum* (MIC = 1,000 *μ*g/mL), *C. neoformans* (MIC = 1,000 *μ*g/mL), and *C. albicans* (CIM = 1,000 *μ*g/mL) and strong activity against *T. mentagrophytes* (MIC = 500 *μ*g/mL) [24].

Bersan et al. [17] also evaluated the anti-microbial activity of *A. gratissima* oil by microdilution against the following microorganisms: *C. albicans* (CBS 562), *Streptococcus sanguis* (ATCC 10556), *Streptococcus mitis* (ATCC 903), *Porphyromonas gingivalis* (ATCC 33277), and *Fusobacterium nucleatum* (ATCC 25586). The *Aloysia* oil significantly inhibited the biofilm growth of *P. gingivalis*, *S. sanguis*, and *S. mitis* (9.0%; *p* ≤ 0.05).

Galvez et al. [20] reported that the essential oil from the aerial parts of *A. gratissima* shows moderate antifungal activity against toxigenic *Fusarium* (MIC = 0.6–1.2 mg/mL) and was mainly inactive against *Aspergillus* species.

In addition to the chemical investigation of the *A. gratissima* oil, Santos et al. [32] studied the variable activity against Gram-positive bacteria (*Bacillus subtilis* CCT 2576, *S. aureus* CCT 2740, and *Streptococcus pneumoniae* ATCC 11733), Gram-negative bacteria (*Salmonella choleraesuis* CCT 4296 and *P. aeruginosa* ATCC 13388), and fungus (*C. albicans* ATCC 10231). The antimicrobial activity varied according to the microorganisms evaluated: the essential oil demonstrated an MIC 0.8 mg/mL against *P. aeruginosa* and MIC 0.6 mg/mL for *S. pneumonia*. The essential oil of the inflorescence showed activity against *P. aeruginosa* (MIC 0.15 mg/mL), *S. pneumoniae* (MIC 0.025 mg/mL), and *C. albicans* (0.02 mg/mL). According to the authors, the essential oil of the inflorescence was more effective against the microorganisms than the essential oil of the leaf, and it was especially pronounced against the Gram-negative bacteria *P. aeruginosa*, the Gram-positive bacteria *S. pneumonia*, and the yeast *C. albicans*.

Regarding the antiprotozoan effect of *A. gratissima*, the study by Garcia et al. [22] reports promising results of the *A. gratissima* oil, acting directly on *Leishmania amazonensis* (WHOM/BR/75/Josefa) parasite by affecting the kinetoplast, plasma membrane, and mitochondrial matrix. The authors correlate these results with the chemical composition of the oil, being guaiol the main compound. They also report that the oil affected the promastigote growth regardless of the dose and obtained inhibitions between 31% and 85% of the parasite growth, resulting in IC50 = 25 *μ*g/mL, after 48 h and IC50 = 14 *μ*g/mL, after 72 h. Garcia et al. [22] also investigated the effect of *A. gratissima* oil on intramacrophage amastigotes load. It was found a reduction in 85% of the amastigote intracellular load (at 2 *μ*g/mL of *A. gratissima* oil, similar to the reduction promoted by 1 *μ*g/mL of amphotericin B).

The authors state that guaiol can be directly metabolized by parasites, generating toxic products, or by host cells, generating a leishmanicidal metabolite. Garcia et al. [22] consider that *A. gratissima* can contribute to the development of new antiprotozoal drugs.

The chemical compound guaiol, widely found in species of the genus *Aloysia*, is sesquiterpenoid alcohol found in medicinal plants, mainly in Cypress and Guaiacum woods. This compound shows antibacterial and antitumor activities [40]. In this sense, Souza and Wiest [11] reported antimicrobial activity of *A. gratissima* against the *Rhodococcus equi* and *Pasteurella* sp. (isolated from the animal clinic) bacteria.

### 3.5. Virucidal Effect

Garcia et al. [21] studied the virucidal activity of *A. gratissima* oil obtained by hydrodistillation against herpes simplex virus type 1 (HSV-1), Junin virus (JUNV), and dengue virus type 2 (DEN-2). Satisfactory results were found for the JUNV, with VC50 values ranging from 52 to 90 ppm. The VC50 against DNA/HSV-1 was 65 ppm; therefore, it is considered less susceptible than the JUNV virus. However, *A. gratissima* oil was not effective against the DEN-2.

### 3.6. Antioxidant Effect

Zeni et al. [29] found promising results related to the antioxidant activity of *A. gratissima* oil. The authors analyzed the lyophilized oil of *A. gratissima* for total polyphenols (TP), total carotenoids (TC), total flavonoids (TF), and sequestering activity of free radicals.

The main compounds found in the oil were lutein (0.30–1.30 mg/g) and trans-b-carotene (0.24–1.86 mg/g). Other compounds were revealed by HPLC, such as ferulic acid (11.57–86.40 mg/100 g), trans-cinnamic acid (7.97–56.92 mg/100 g), and p-coumaric acid (0.00–16.06 mg/100 g), which were the most abundant phenolic acids in the studied samples. Caffeic, chlorogenic, gallic, vanillic, and protocatechuic acids were also found in the samples. As mentioned by the authors, climatic factors can significantly influence the accumulation of phenolic acid in the tissues of *A. gratissima*, indicating that studies on biological effects of the extracts/oils should take into account the influence of seasonality on the metabolic profile of this species.

Regarding the inhibition of free radicals, the author describes that the extracts depend on the concentration and synergistic effects of phytonutrients and may even be affected by other components involved in the cell complex, such as enzymatic components and glutathione. Furthermore, Zeni et al. [29] observed that the polyphenolic profiles revealed that some phenolic acids are present in a higher concentration than others, according to the month of the plant harvest, presenting an equilibrium throughout the seasons. Thus, it is indicated that the phenolic compounds significantly contributed to the antioxidant capacity of *A. gratissima*, with *R*^2^ = 0.9489 (*p* < 0.05), revealing the EC50 value of 1 mg/mL. Zeni et al. [29] also report that the antioxidant activity of *A. gratissima* is directed towards cellular lipid environments, such as organelle membranes.

### 3.7. Effects on the Central Nervous System

Zeni et al. [14] studied the *in vivo* antidepressant-like activity of *A. gratissima* oil obtained by steam dragging the leaves. The authors used the forced swimming test (FST) and the tail suspension test (TST) in mice. Their results show a reduction in the immobility time of mice treated with *A. gratissima* oil in both tests. Also, they demonstrated that the antidepressant-like activity occurs through the inhibition of N-methyl-D-aspartate (NMDA) receptors. Therefore, the authors suggest that this species has the potential to develop new drugs with antidepressant effects.

Benovit et al. [6] demonstrated the anesthetic and sedative properties of *A. gratissima* using silver catfish as an animal model. The authors performed a bio-guided fractionation of the essential oil and demonstrated the importance of (+)-spathulenol in its sedative and anesthetic properties.

### 3.8. Toxicological Evaluation of *A. gratíssima*

Garcia et al. [22] evaluated the toxicity of *A. gratissima* oil against host macrophages by the XTT method. The phagocytosis capacity of macrophages was not inhibited by the oil. Nevertheless, the authors report that there was an increase of around 50% of the phagocytosis of macrophages after treatment with *A*. *gratissima* oil.

In the *in vivo* toxicological evaluation of *A. gratissim*a, Souza et al. [26] and Zeni et al. [29] reported the use of Swiss mice treated with 2,000 mg/kg (p.o.). During the 14 days of observation, there was no mice death in both studies.

Souza et al. [26] reported that there was intense sedation 45 minutes after the oral administration, but after this period, the animals fed normally and did not present locomotor changes. During the 14 day observation period, the authors report that there was no difference in the food intake and body weight between the group treated with the supercritical extract of *A. gratissima* leaves and the control group. Also, the oral administration of supercritical extract of *A. gratissima* leaves did not cause changes in the relative weight of the brain, liver, kidneys, lung, heart, and thymus. However, there was a significant decrease in the relative weight of the spleen and adrenal glands in the supercritical-extract-treated mice. These findings may indicate a sign of toxicity that should be further investigated in a repeated dose toxicity test.

It is important to note that there is a difference between the extraction methods performed in the studies by Zeni et al. [29] (hydroalcoholic extract) and by Souza et al. (extraction by SCCO_2_). Nevertheless, the results of both studies agree with each other regarding the findings of the plant material toxicity.

## 4. Conclusions

South America stands out in scientific studies with the species *A. gratissima*. Terpenes are the main chemical compounds present in *A. gratissima*, being Guaiol, Pinocanfone, *ß*-elemene, *ß*-caryophyllene, spathulenol, trans-pinocamphone, and trans-pinocarveol acetate the main ones. It is observed that the extraction method used in the studies does not impact significantly in the chemical composition of the extracts; however, the period of harvest does. Taken together, the data found in the 21 reviewed studies suggest that *A. gratissima* has promising potential to be a source of anti-inflammatory, antinociceptive, antimicrobial, and/or antidepressant compounds.

## Figures and Tables

**Table 1:** Description of plant compounds and research aspects of the studies included in the systematic review.

Reference/year	Part of the plant	Extraction method	Chemical composition		Biological activities
Benovit et al. (2015) [6]	Leaves	Hydrodistillation/Clevenger	1,8-Cineole (18.54%), sabinense (9.5%), guaiol (6.79%), and bicyclogermacrene (5.12%)	*In vivo*	Sedative/anesthetic
Arze et al., (2013) [16]	Leaves	Hydrodistillation/Clevenger	Sabinene (30–35%), *ß*-pinene (8–10%), and *ß*-caryophyllene (∼8%)	—	—
Bersan et al., (2014) [17]	Leaves	Hydrodistillation/Clevenger	E-Pinocamphone (16.07%), *ß*-pinene (12.01%), guaiol (8.53%), E-pinocarveol acetate (8.19%), and *α*-caryophyllene (7.19%)	*In vitro*	Antimicrobial
Consolini et al. (2011) [18]	Leaves	Decoction	—	*In vivo*	Antispasmodic
Freires et al. (2015) [19]	Leaves	Hydrodistillation/Clevenger	Guaiol (29.63%), trans-pinocarveol (13.16%), bulnesol (11.79%), and myrtenol (5.3 1%)	*In vitro*	Antimicrobial
Galvez et al. (2020) [20]	Leaves	Hydrodistillation/Clevenger	*β*-Thujone (36.1 ± 0.1%), *α*-thujone (32.2 ± 0.2%), 1,8-cineol (10.7 ± 0.1%), and sabinene (6.2 ± 0.2%)	*In vitro*	Antifungal
Garcia et al. (2003) [21]	Leaves	Hydrodistillation/Clevenger	Caryophyllene oxide (15.84%), cadinol (17.37%), chrysanthenyl acetate (5.61%), limonene oxide (5.29%), and *ß*-caryophyllene (4.77%)	*In vivo*	Virucidal—JUNV e HSV-1
Garcia et al. (2018) [22]	Leaves and fruits	Hydrodistillation/Clevenger	1,8-Cineole (17.64%) and guaiol (10.33%)	*In vivo*	Antileishmanial activity
Hister et al. (2009) [23]	Leaves	Hydrodistillation/Clevenger	—	*In vitro*	Inhibition of tumor cells growth
Ricciardi et al. (2006) [9]	Leaves	Hydrodistillation/Clevenger	*β* –Caryophyllene (25.1%), germacrene D (10.1%), bicyclogermacrene (12.6%), and *α*-thujone (10.7%)	—	—
Santos et al. (2015) [24]	Leaves	Hydrodistillation/Clevenger	1,8-Cineole (13.7%), germacrene D (13.4%), *ß*-caryophyllene (12.7%), and *ß*-pinene (11.7%)	*In vitro*	Antibacterial and antifungal
Soler (1986) [25]	Leaves	—	*β*-Pinene (8%)	—	—
Souza and Wiest (2007) [11]	—	Decoction	—	*In vitro*	Bacteriostatic
Souza et al. (2020) [13]	Leaves	Supercritical fluid with carbon dioxide	Guaiol (18.50%) and pinocamphone (11.40%)	*In vivo*	Anti-inflammatory
Souza et al. (2020) [26]	Leaves	Supercritical fluid with carbon dioxide	Guaiol (18.50%) and pinocamphone (11.40%)	*In vivo*	Antinociceptive
Trovati et al. (2009) [27]	Leaves	Hydrodistillation/Clevenger	Isopinocamphone (*trans*-3-pinanone; 25.4%), limonene (15.1%), and guaiol (12.7%)	—	—
Vandresen et al. (2010) [28]	Leaves	Cold maceration/ethanol	*α*-bisabolol	*In vitro*	Antibacterial and Antiedematogenic effect
Zeni et al. (2011) [14]	Leaves	Hydrodistillation/Clevenger	—	*In vivo*	Antidepressant-like effect
Zeni et al. (2013) [29]	Leaves	Decoction	Ferulic acid (11.57–86.40 mg/100 g), trans-cinnamic acid (7.97–56.92 mg/100 g), and p-coumaric acid (0.00–16.06 mg/100)	*In vivo* and *in vitro*	Toxicity and antioxidant
Zeni et al. (2013) [30]	Stems and leaves	Decoction	—	*In vivo*	Antidepressant-like effect
Da Silva et al. (2006) [31]	Leaves	Cold maceration/ethanol	Guaiol, bisabolol, and spathulenol	—	—
Santos et al. (2013) [32]	Leaves	Hydrodistillation/Clevenger	Transpinocarveyl acetate (17.6%), trans-pinocamphone (16.3%), and guaiol (11.5%)	*In vitro*	Antibacterial and antifungal

## Data Availability

The data supporting this systematic review are from previously reported studies and data sets, which have been cited.
